# Overcoming the plasticity of plant specialized metabolism for selective diterpene production in yeast

**DOI:** 10.1038/s41598-017-09592-5

**Published:** 2017-08-18

**Authors:** Codruta Ignea, Anastasia Athanasakoglou, Aggeliki Andreadelli, Maria Apostolaki, Minas Iakovides, Euripides G. Stephanou, Antonios M. Makris, Sotirios C. Kampranis

**Affiliations:** 1 Department of Plant and Environmental Sciences, University of Copenhagen, Thorvaldsensvej 40, 1871 Frederiksberg C, Denmark; 2 Department of Medicine, University of Crete, P.O. Box 2208, Heraklion, 71003 Greece; 3 Institute of Applied Biosciences – Centre for Research and Technology Hellas (INAB-CERTH), P.O. Box 60361, Thermi, 57001 Thessaloniki, Greece; 4 Department of Chemistry, University of Crete, P.O. Box 2208, Heraklion, 71003 Greece

## Abstract

Plants synthesize numerous specialized metabolites (also termed natural products) to mediate dynamic interactions with their surroundings. The complexity of plant specialized metabolism is the result of an inherent biosynthetic plasticity rooted in the substrate and product promiscuity of the enzymes involved. The pathway of carnosic acid-related diterpenes in rosemary and sage involves promiscuous cytochrome P450s whose combined activity results in a multitude of structurally related compounds. Some of these minor products, such as pisiferic acid and salviol, have established bioactivity, but their limited availability prevents further evaluation. Reconstructing carnosic acid biosynthesis in yeast achieved significant titers of the main compound but could not specifically yield the minor products. Specific production of pisiferic acid and salviol was achieved by restricting the promiscuity of a key enzyme, CYP76AH24, through a single-residue substitution (F112L). Coupled with additional metabolic engineering interventions, overall improvements of 24 and 14-fold for pisiferic acid and salviol, respectively, were obtained. These results provide an example of how synthetic biology can help navigating the complex landscape of plant natural product biosynthesis to achieve heterologous production of useful minor metabolites. In the context of plant adaptation, these findings also suggest a molecular basis for the rapid evolution of terpene biosynthetic pathways.

## Introduction

A unique feature of plants is their inherent phenotypic plasticity. Being immobile organisms, plants cannot move away from unfavorable conditions. As a result, they respond to environmental changes by altering their morphology, physiology and metabolism. Metabolic plasticity, in particular, involves the synthesis of an astonishing number of specialized metabolites, also known as natural products, which are responsible for plant defense and adaptation, mediating multipurpose interactions among organisms^1–5^. Plants have developed complex metabolic pathways that enable them to rapidly and dynamically change the profile of specialized metabolites produced in order to adapt to diverse and fluctuating environmental factors. Many of these compounds have important applications as pharmaceuticals, flavors, fragrances or biofuels^6–8^. However, exploitation of plant natural products is frequently hindered by metabolic plasticity. Potential pharmacologically or industrially relevant metabolites are only produced in minute amounts, under specific conditions or environmental stimuli, or in complex mixtures of structurally related compounds. Thus, achieving efficient biotechnological production of compounds of interest requires the development of approaches that navigate the complex landscape of plant specialized metabolism to bypass the limitations imposed by metabolic plasticity.

**Figure 1:**
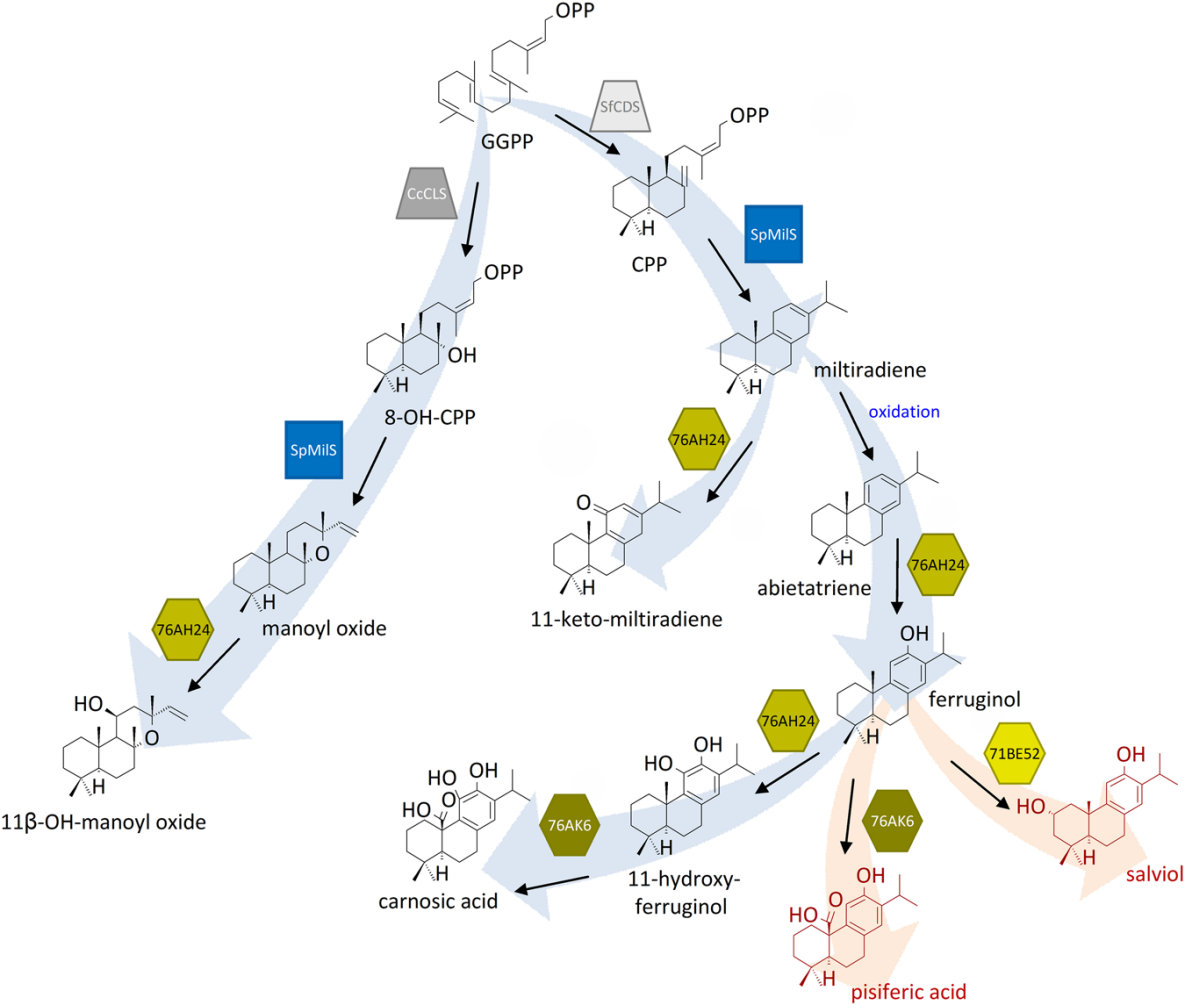
Diagrammatic representation of the biosynthetic pathway of labdane-type diterpenes and its reconstruction in yeast. Starting from the common precursor geranylgeranyl diphosphate (GGPP), different class II diTPSs synthesize copalyl diphosphate (CPP) or 8-hydroxy-copalyl diphosphate (8-OH-CPP). Miltiradiene synthase (MilS) can accept either CPP or 8-OH-CPP to produce miltiradiene or manoyl oxide, respectively (refs 11, 20). Subsequently, the promiscuous enzyme, CYP76AH24, oxidizes miltiradiene, abietatriene, ferruginol and manoyl oxide, to synthesize the corresponding oxygenated molecules in position C-12 or C-11 (the biosynthetic pathways are described in details in the main text). Different CYPs perform additional oxidations to produce carnosic and/or pisiferic acid (CYP76AK6), or salviol (CYP71BE52). Arrows describe the fluxes through the yeast reconstructed pathway before (blue arrows) or after engineering (red arrows) for the specific production of pisiferic acid or salviol (Figure produced using ChemDraw Professional 15.1 and Microsoft Powerpoint).

The mechanisms underlying plant natural product plasticity rely on the promiscuity of the corresponding biosynthetic enzymes. Most enzyme groups involved in specialized metabolism exhibit relaxed substrate or product specificity. This is particularly true in the case of terpenoids, the largest class of plant natural products^9^. Terpene synthases use a handful of simple precursors to generate a broad range of terpenoid skeletons. For example, γ-humulene synthase synthesizes 52 different sesquiterpenes from farnesyl diphosphate (FPP)^10^, while several class I diterpene synthases (diTPSs) accept alternative diterpene diphosphate substrates with similar efficiency to produce different compounds^11–16^. Cytochrome P450 monooxygenases (CYPs), key enzymes for terpenoid structural diversity, are also quite promiscuous. Several multi-substrate and multi-functional CYPs have been identified, including enzymes of the CYP720B subfamily, involved in diterpene resin acids biosynthesis in gymnosperms^17, 18^, and members of the CYP76AH and CYP76AK subfamilies, which participate in carnosic acid biosynthesis in sage and rosemary^19–22^ (Fig. 1) or tanshinone biosynthesis in the Chinese medicinal plant *Salvia miltiorrhiza* ^23^. Achieving efficient production of compounds of interest in a heterologous host may thus require the engineering of the enzymes involved to generate dedicated biocatalysts^11^. The same also applies for the isolation of desirable products from large scale combinatorial biosynthesis efforts^16^.

Carnosic acid is a potent antioxidant present in high amounts in rosemary and sage^24^. This diterpenoid is synthesized, together with a group of structurally related compounds, through a complex biosynthetic pathway (Fig. 1 and ref. 19). Carnosic acid-related diterpene biosynthesis starts from the universal diterpene precursor geranylgeranyl diphosphate (GGPP). This is cyclized through the action of class II and class I diterpene synthases to the basic labdane-type skeleton of miltiradiene, which is further oxidized to abietatriene (Fig. 1). In the sage *S. pomifera*, the final steps of this pathway involve the enzymatic activity of three CYPs, CYP76AH24, CYP71BE52 and CYP76AK6. CYP76AH24 is a bifunctional enzyme that catalyzes the synthesis of ferruginol by oxidation of abietatriene at C-12 and subsequently adds a second hydroxyl group at position C-11 of ferruginol to produce 11-hydroxy-ferruginol^19^. The same enzyme accepts additional substrates, such as miltiradiene and manoyl oxide, to produce 11-keto-miltiradiene (Fig. 1 and ref. 19) and 11β-hydroxy-manoyl oxide (Fig. 1 and ref. 20), respectively. CYP71BE52 hydroxylates ferruginol at C-2*α* to produce salviol^19^, a compound also found in the bioactive extract of *S. miltiorrhiza* roots^25, 26^. Additional biosynthetic steps involve oxidation of ferruginol and 11-hydroxy-ferruginol to produce pisiferic acid and carnosic acid (Fig. 1). Pisiferic acid, which is an antibacterial^27–29^ and antifungal^30, 31^ agent, is produced from ferruginol by successive oxidations at position C-20 catalyzed by CYP76AK6^19^ (Fig. 1). However, the same enzyme is also responsible for the synthesis of carnosic acid from 11-hydroxy-ferruginol (Fig. 1). This highly complex pathway organization is an important hurdle in the study or exploitation of individual compounds in the group, particularly those present in lower abundance, as their isolation from a natural source is highly challenging. Transplanting the pathway leading to specific compounds in a heterologous host is a viable alternative, but the promiscuity of the enzymes involved, essential for maintaining the plasticity of the natural system, is limiting.

In this report, we initially engineered yeast cells for the production of carnosic acid and achieved significant improvement in yield compared to our previous efforts^19^. Still, in the improved system, production of the pharmacologically interesting minor products pisiferic acid and salviol was limited and always accompanied by high levels of undesirable by-products. To enable production of these compounds, we set out to redirect the pathway away from carnosic acid (Fig. 1). Using protein engineering, we altered the specificity of a central enzyme in this biosynthetic pathway, CYP76AH24. A single amino acid substitution restricted the substrate specificity of CYP76AH24 and gave rise to a dedicated ferruginol synthase. Using this CYP variant in yeast, we achieved the complete biosynthetic shift from carnosic acid to pisiferic acid and we improved the specificity of salviol synthesis by reducing the undesirable by-products 11-keto-miltiradiene and 11-hydroxy-ferruginol. These results provide an example of how protein and metabolic engineering can be combined to overcome the plasticity of plant specialized metabolism for the heterologous production of useful metabolites that are only produced in minor amounts in the plant.

## Results

### Optimization of carnosic acid production in yeast

**Figure 2:**
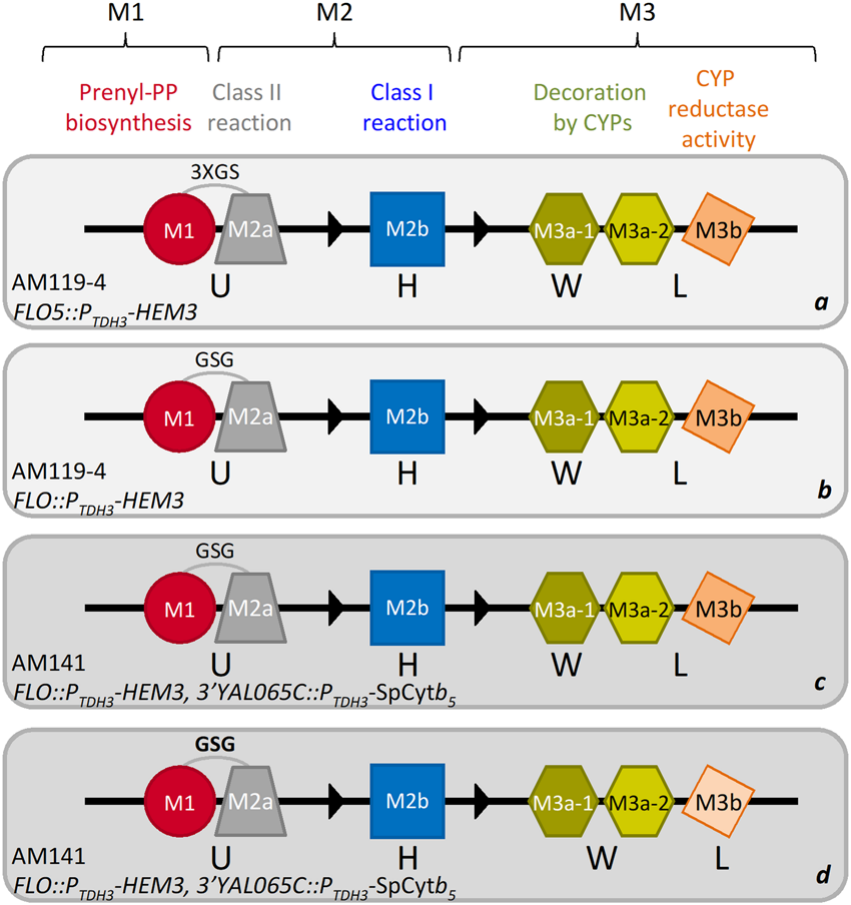
Depiction of the engineered yeast platform for production of carnosic acid-related diterpenes. The modular yeast platform previously developed in AM119-4 strain (panel ***a***, light grey background; ref. 20) was optimized for carnosic acid production in three steps, including engineering of a shorter linker (GSG) between the M1 and M2a parts (panel ***b***), chromosomal integration of SpCyt*b*_5_ in strain AM141 (dark grey background) (panel ***c***), and modulation of CPR expression by switching from the strong inducible promoter P_GAL1_ (dark orange) to the weaker constitutive P_TPI1_ promoter (light orange) (panel ***d***).

In our previous efforts, we developed a modular yeast diterpene production platform to facilitate biosynthetic pathway elucidation through rapid screening of candidate genes obtained from transcriptomic analysis^19, 21^. In this platform, described diagrammatically in Fig. 2 panel a, module M1 is responsible for GGPP synthesis, module M2 (consisting of two submodules, M2a and M2b) includes the steps leading to the formation of the basic terpene scaffold, and module M3 encompasses the events achieving the decoration/modification of the basic skeleton. In the configuration used for carnosic acid pathway elucidation^19^, M1 employed a yeast farnesyl diphosphate synthase variant (Erg20p(F96C)) that functions as a highly efficient GGPP synthase^32^. Submodule M2a, responsible for copalyl diphosphate (CPP) formation, involved *S. fruticosa* copalyl diphosphate synthase (SfCDS), and submodule M2b, responsible for terpene cyclization, used *S. pomifera* miltiradiene synthase (SpMilS) to convert CPP to miltiradiene. In M3, terpene oxidation events were catalyzed by various CYPs (M3a), assisted by a cytochrome P450 reductase (CPR; M3b) (Fig. 2, panel *a*).Strain AM119-4 (Table 1), engineered for efficient production of oxidized diterpenes^20^, was employed as chassis. In this configuration, carnosic acid titer reached 1 mg/L of yeast culture^19^.

**Table 1:** List of *S. cerevisiae* strains used.

Strain	Genotype	Source
AM119-4	(Mat a/α, P_Gal1_-*HMG2*(K6R)::HOX2, *ura3, trp1*, *his3*, P_TDH3_-*HMG2*(K6R)*X*2-::*leu2 ERG9*/*erg9*, *ubc7/UBC7*, *ssm4/SSM4, mct1/MCT1, whi2/WHI2, gdh1/GDH1*, P_TDH3_ *-HEM3::FLO5*.	ref. 20
AM141	Mat a/α, P_Gal1_-*HMG2*(K6R)::HOX2, *ura3, trp1*, *his3*, P_TDH3_-*HMG2*(K6R)*X*2-::*leu2 ERG9*/*erg9*, *ubc7/UBC7*, *ssm4/SSM4, mct1/MCT1, whi2/WHI2, gdh1/GDH1*, P_TDH3_ *-HEM3::FLO5*, P_TDH3_ *-SpCytb*_*5*_ *::3*′*YAL065C*	this study

**Figure 3:**
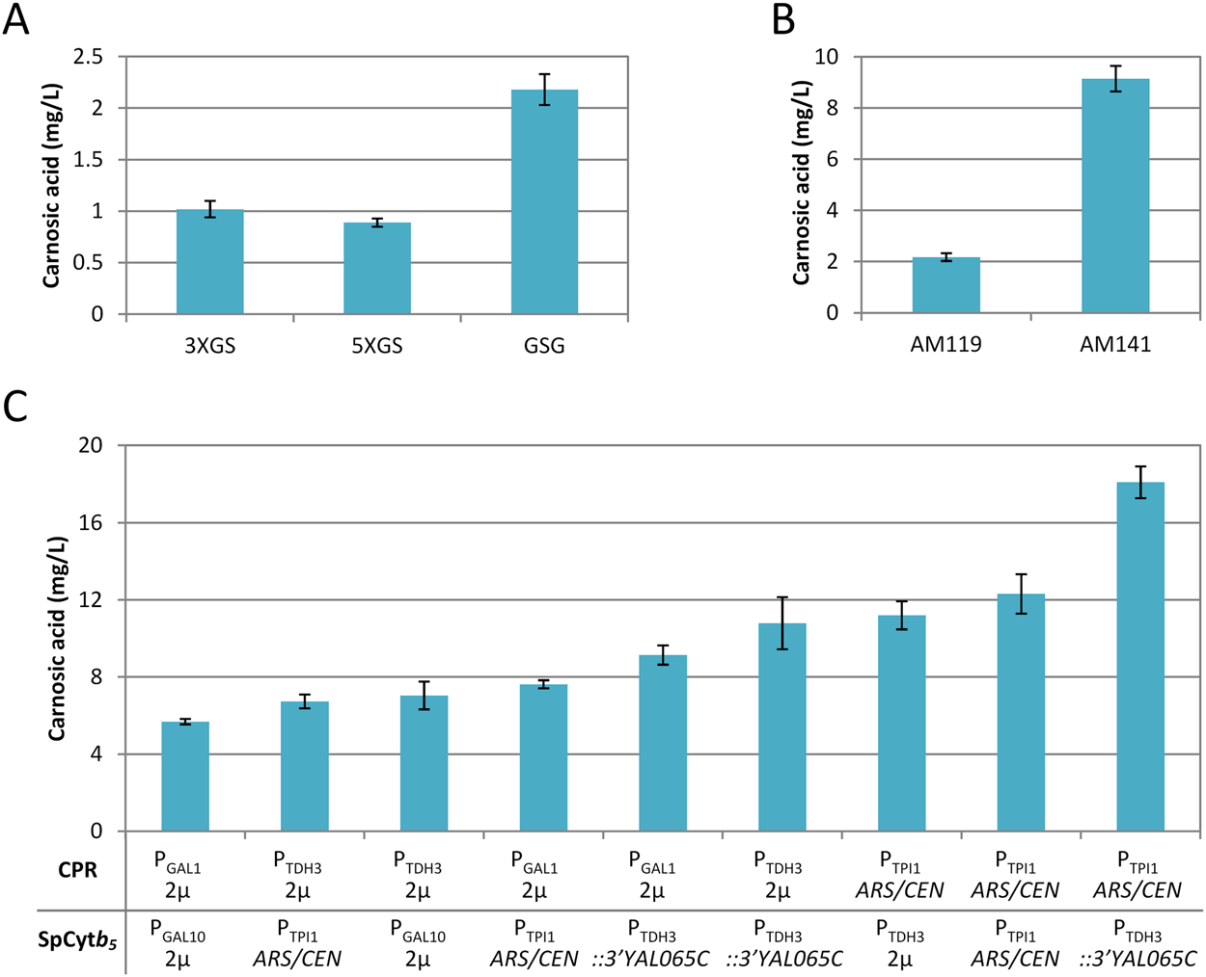
Optimization of carnosic acid production in yeast. (**A**) Production of carnosic acid was evaluated using linkers of different size between the M1 and M2a module-specific parts. 2-fold improvement was achieved using the GSG tripeptide linker (M1-GSG-M2a). (**B**) Expression of SpCyt*b*_5_ from a single chromosomal copy (strain AM141) further increased the CA yield by 4-fold. (**C)** Balancing the CPR/SpCyt*b*_5_ expression further increased carnosic acid production to 18 mg/L. CPR and SpCyt*b*_5_ were expressed from chromosomal integration (*::3*′*YAL065C*), from a low copy-number plasmid (*ARS/CEN*), or from a high copy number plasmid (2μ), under the control of constitutive (P_TPI1_ or P_TDH3_) or inducible (P_GAL1_ or P_GAL10_) promoters of different strength (P_TPI1_ – medium or P_TDH3_, P_GAL1_ or P_GAL10_ – strong).

To improve carnosic acid production, a series of modifications were introduced in this platform. To facilitate metabolic channeling, the starting configuration employed a fusion between the M1 and M2a specific parts (Erg20p(F96C) and SfCDS). This fusion was based on a linker of three Gly-Ser repeats (3xGS) between the two partners^19^. Previous studies have shown that the length of the linker may influence the efficiency of substrate channeling and fusion protein stability, impacting heterologous production of target compounds^33^. To optimize linker length, we constructed and tested two fusions with linkers of different size. One containing five GS repeats (SfCDS-5xGS-Erg20p(F96C)) and one containing the GSG tripeptide (SfCDS-GSG-Erg20p(F96C)). Compared to the original 3xGS fusion, carnosic acid production in yeast cells engineered to express the two new fusion proteins was slightly reduced in the case of the 5xGS linker, but improved by 2-fold when the shorter GSG linker was used (Fig. 2 panel ***b***, Fig. 3A and Table 2).

**Table 2:** Overview of carnosic acid yield improvement.

strain	protein expressed	linker	carnosic acid yield (mg/L of culture)	fold improvement (from previous step)	fold improvement (total)
AM119-4	CYP76AH24wt	3XGS	1.02 ± 0.08	—	—
AM119-4	CYP76AH24wt	GSG	2.18 ± 0.15	2.13	2.13
AM141	CYP76AH24wt	GSG	9.15 ± 0.50	4.19	9.33
AM141	CYP76AH24wt + CPR(P_TPI1_)	GSG	18.09 ± 0.82	1.97	17.73

Previous studies have shown that the efficiency of some CYP-catalyzed oxidations in yeast is improved by the presence of cytochrome *b*5 (Cyt*b*_5_)^34–36^ that may function as a partner or modulator in CYP/CPR complexes. To evaluate the effect of Cyt*b*_5_ in carnosic acid biosynthesis in yeast, we searched our *S. pomifera* trichome-specific transcriptomic dataset^21^ and identified one SpCyt*b*_5_ transcript present in high abundance (indicated by its FPKM value). This gene (DNA sequence in Supplementary Information) was amplified from *S. pomifera* trichome-derived cDNA and integrated at the 3′-end of the YAL065C locus of strain AM119-4^20^ under the control of the strong constitutive promoter P_TDH3_, generating strain AM141 (Table 1). Expression of SpCyt*b*_5_ in AM141 resulted in approximately 4-fold yield improvement over the parental strain, AM119-4 (Fig. 2 panel c and Fig. 3B, Table 2), reaching 9.15 mg/L carnosic acid.

High-level expression of cytochrome P450 reductase (CPR) may lead to inefficient interactions with its CYP partners resulting in significant leak of electrons and an overall increase of oxidative stress in engineered heterologous production systems^37^. On the other hand, low CPR expression may not provide CYP enzymes with adequate reducing equivalents for efficient function. Since CPR expression could be a rate-limiting factor for CYP activity, we set out to balance co-expression of CPR, CYPs and Cyt*b*_5_ in carnosic acid-producing yeast cells. To enable introduction of a new part into the existent modular platform, we initially generated a pESC-based construct expressing two CYPs (CYP76AH24 and CYP76AK6) under tryptophan selection. Subsequently, we engineered and analyzed AM141 derived yeast cells expressing CPR and Cyt*b*_5_ from a combination of high and low copy number plasmids (containing the 2μ or ARS/CEN origin of replication, respectively) under promoters of varying strength. CPR was evaluated using the strong, inducible P_GAL1_ promoter, the strong constitutive P_TDH3_ promoter, or the medium strength constitutive promoter P_TPI1_ (Fig. 2 panel ***d*** and Fig. 3C, Table S1). Cyt*b*_5_ expression was varied between a high copy number plasmid (pESC) driven by the strong inducible promoter P_GAL10_, a single chromosomal integration under the P_TDH3_ promoter, or from a low copy number plasmid (pYX143) under the constitutive promoter P_TPI1_ (Fig. 2 panel ***d*** and Fig. 3C, Table S1). The highest production of carnosic acid, reaching 18 mg/L (almost 2-fold improvement over the previous intervention), was achieved in yeast cells expressing SpCyt*b*_5_ only from a single chromosomal integration (AM141 strain) and CPR from a low copy number plasmid under the control of the P_TPI1_ constitutive promoter (Fig. 2 panel ***d*** and Fig. 3C, Tables 1 and 2). The growth rate of all strains tested in Fig. 3 was similar, suggesting that the observed titer improvement was not due to higher biomass yield achieved as a result of lowering the metabolic burden of CPR or SpCyt*b*_5_ expression.

### Engineering the specificity of CYP76AH24

**Figure 4:**
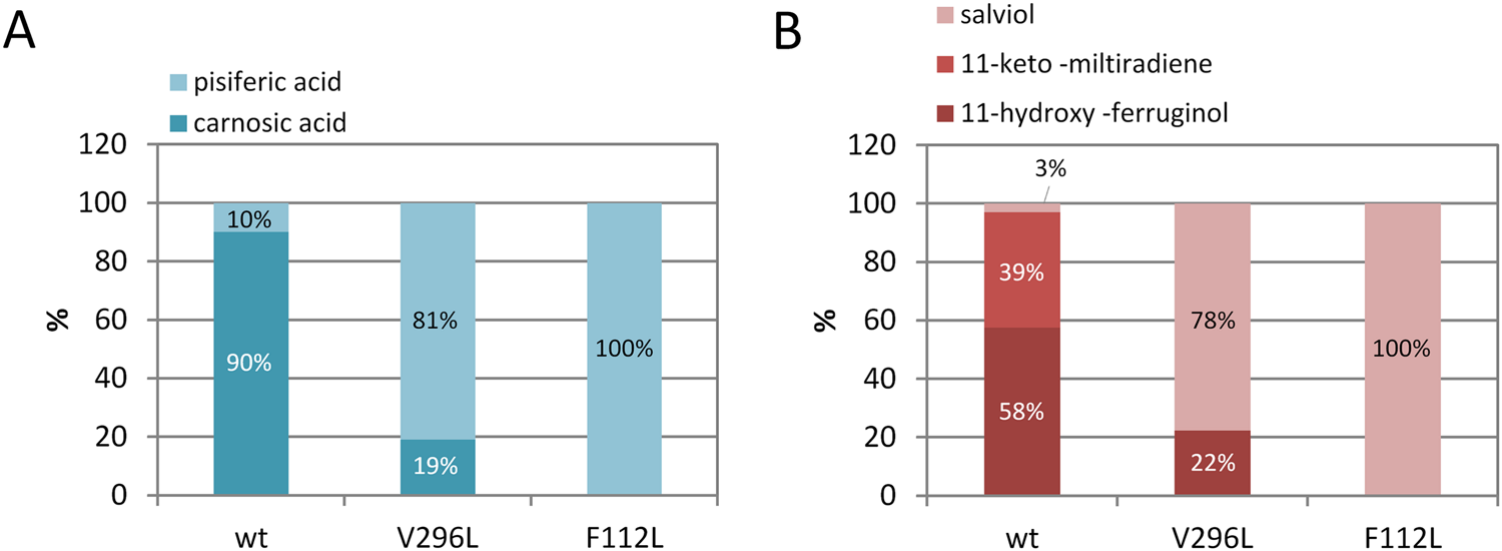
Redirecting biosynthetic fluxes towards specific compounds by engineering CYP76AH24 specificity. (**A**) Biosynthetic shift from carnosic acid to pisiferic acid production in yeast cells. (**B**) Abolishing undesirable production of 11-keto-miltiradiene and 11-hydroxy-ferruginol production in yeast to favor biosynthesis of salviol.

Despite achieving significant improvement in carnosic acid titers using these interventions, production of the minor metabolites pisiferic acid and salviol was still limited. Like carnosic acid, pisiferic acid is also formed by oxidation of C-20 by CYP76AK6. For pisiferic acid synthesis, CYP76AK6 acts on ferruginol, while for carnosic acid it acts on 11-hydroxy-ferruginol, both produced by CYP76AH24. However, when both CYP76AK6 and CYP76AH24 are present, yeast cells produce almost exclusively carnosic acid (carnosic acid/pisiferic acid ratio 10:1; Fig. 4A, Table S2, and ref. 19). This is because the ability of CYP76AK6 to catalyze oxidation of 11-hydroxy-ferruginol compared to ferruginol is different, favoring carnosic acid biosynthesis^19^. Similarly, when salviol production was reconstructed using CYP76AH24 and CYP71BE52 in the improved yeast platform described above, salviol titers reached 1.1 mg/L but salviol was not the main product (Fig. 4B, Table S2). Due to the ability of CYP76AH24 to also oxidize C-11 of miltiradiene and ferruginol, salviol production in this system was dominated by 13.5 mg/L of 11-keto-miltiradiene and around 20 mg/L of 11-hydroxy-ferruginol (Fig. 4B, Table S2).

Aiming to engineer efficient production of the specific minor compounds, pisiferic acid and salviol, we undertook a protein engineering effort to overcome the plasticity of the carnosic acid pathway by altering the catalytic specificity of key enzymes in the pathway. Examination of the complex biosynthetic scheme of carnosic acid-related diterpenes (Fig. 1) reveals that although CYP76AK6 catalyzes the synthesis of both carnosic acid and pisiferic acid with kinetic parameters favoring carnosic acid biosynthesis^19^, this enzyme does not present an immediate target for mutagenesis for directing the flux of the pathway towards pisiferic acid. Rather, CYP76AH24 presents a better point for intervention, as it lies in a central node of the pathway after which this bifurcates to an 11-hydoxy-ferruginol branch leading to carnosic acid and a ferruginol branch that leads to pisiferic acid or salviol (Fig. 1). CYP76AH24 is a bifunctional enzyme producing the precursor for both these branches, and if it could be tailored to produce predominantly ferruginol this would redirect the pathway towards the desirable minor compounds.

**Figure 5:**
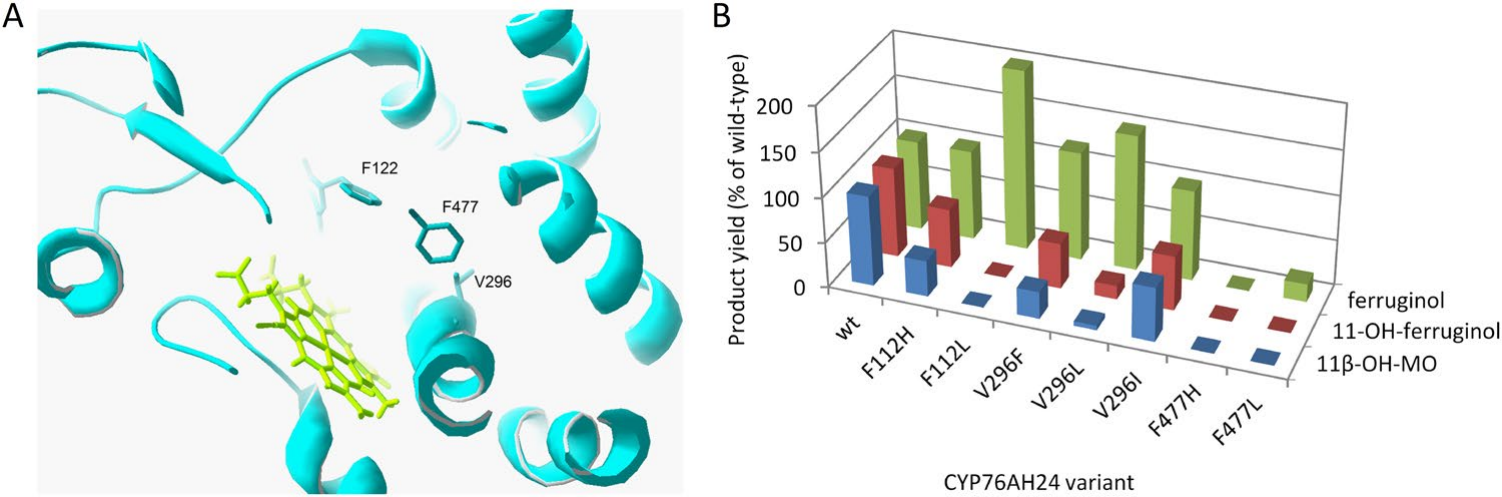
Engineering CYP76AH24 specificity. (**A**) Model of CYP76AH24 showing the residues selected for mutagenesis (F112, V296 and F477). Graphic produced by DeepView (Swiss-PdbViewer). (**B**) Product profile of selected mutants. Products include: ferruginol (green), 11-hydroxy-ferruginol (red) and 11*β*-hydroxy-manoyl oxide (blue). Two variants, F112L and V296L, showed substrate selectivity and improved catalytic activity. Product titers for each of the three substrates were normalized based on the corresponding wild-type CYP76AH24 yield listed in Table S1 and considered as 100%.

Following extensive structural template search, five models of CYP76AH24 were built, based on the structural data of CYP17A1 (PDB ID: 4nkv), CYP2A6(N297Q/I300V) (PDB ID: 2pg7), CYP17A2 (PDB ID: 4r21), CYP3A4 (PDB ID: 4ny4), and CYP2A13 (PDB ID: 3t3s), using Swiss-model (swissmodel.expasy.org). The model based on CYP17A1 displayed the best quality scores (QMEAN = −2.34, GQME = 0.65) and was selected for further analysis (Fig. 5A). It was assumed that to achieve the desired shift in regio-specificity, only a small change in the positioning of the substrate that takes C-11 further away from the heme-bound oxygen, but retains optimal positioning of C-12, would be sufficient. The amino acids that line the active site cavity were mapped and three residues located at key positions shaping the active-site contour where highlighted. These were F112, V296 and F477. Aiming to make only minor adjustments to the geometry of the active site, a minimal mutant library was constructed by substituting each of these amino acids with hydrophobic residues of somewhat larger or smaller size. Thus, F112 was substituted with H or L, V296 with F, L or I, and F477 with H or L.

### Development of a dedicated ferruginol synthase

**Figure 6:**
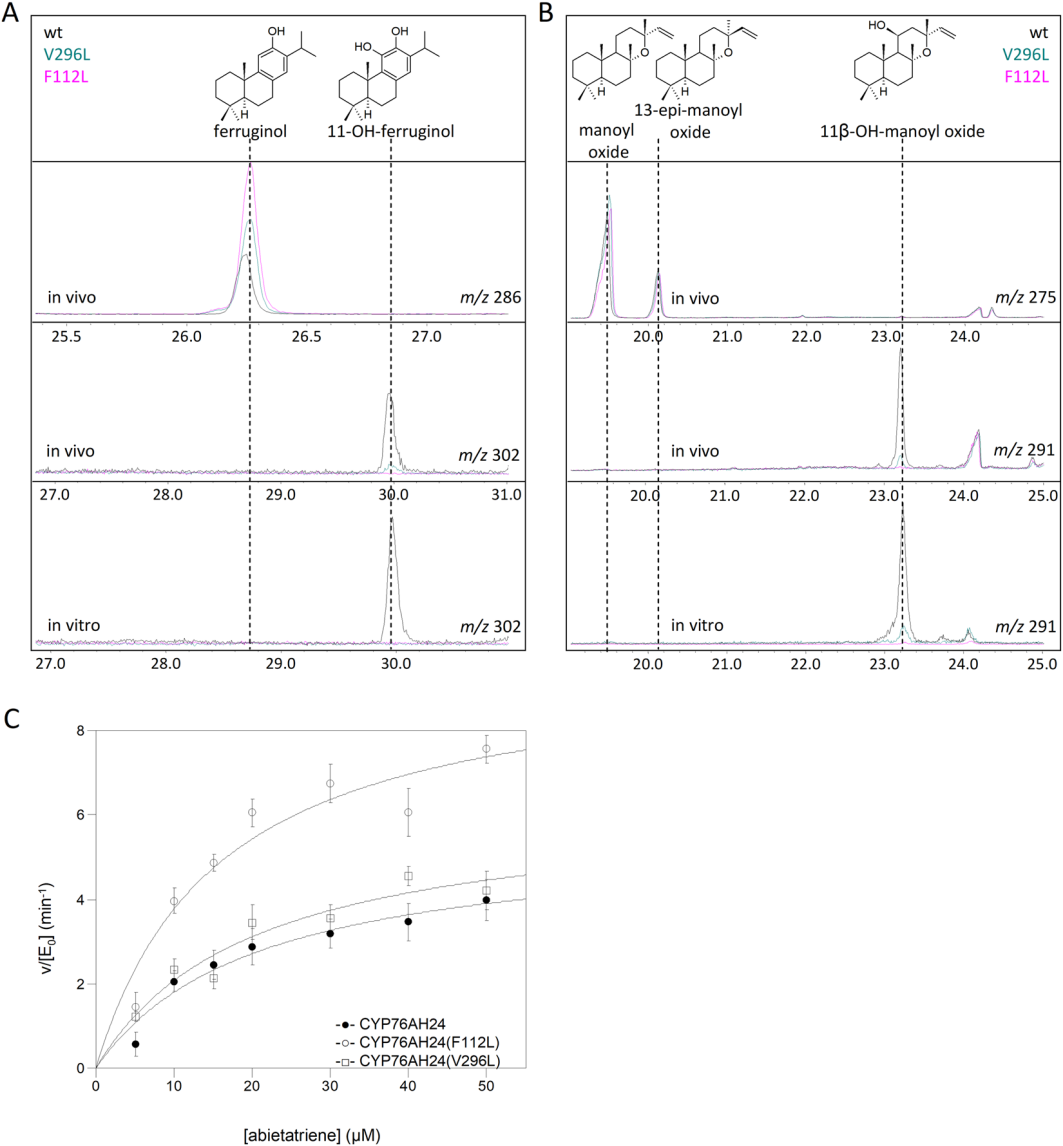
Engineering the specificity of CYP76AH24. (**A)** Evaluation of CYP76AH24 variants in miltiradiene producing yeast cells (V296L (teal) and F112L (pink) versus wt (black)). (**B)** Evaluation of the same CYP76AH24 variants in manoyl oxide producing yeast cells. (**C)** Steady-state kinetic analysis of the oxidation of abietatriene by CYP76AH24 variants V296L (open squares) and F112L (open circles) versus wt (closed circles).

The resulted library was evaluated in yeast cells producing miltiradiene or manoyl oxide and the results of the screening are summarized in the bar chart presented in Fig. 5B. Two variants exhibiting enhanced oxidation of abietatriene at C-12 were identified. These two mutations, F112L and V296L, were also found to improve the substrate specificity of CYP76AH24 by suppressing its ability to oxidize different other substrates, including ferruginol, miltiradiene and manoyl oxide, at position C-11. When expressed in yeast cells, CYP76AH24(V296L) produced 1.5 times more ferruginol, but 8-fold less 11-hydroxy-ferruginol and 20-fold less 11β-hydroxy-manoyl oxide than the wild-type enzyme (Fig. 6A and B, Table S2). No oxidation of miltiradiene was detected in yeast cells using this mutant. CYP76AH24(F112L) catalyzed ferruginol production in yeast 2 times more efficiently than the wild-type enzyme, while production of 11-hydroxy-ferruginol was abolished (Figs 4A and 6A, Table S2). Moreover, this variant did not oxidize miltiradiene or manoyl oxide in the yeast system (Fig. 6A and B). *In vitro* enzymatic assays using yeast microsomal preparations of CYP76AH24(V296L) and CYP76AH24(F112L) with ferruginol or manoyl oxide as substrate and NADPH as co-factor confirmed the reduced ability (or complete lack of) of these variants to oxidize the provided substrates (Fig. 6A and B). Kinetic analysis of the oxidation of abietatriene by CYP76AH24(V296L) and CYP76AH24(F112L) revealed biocatalysts with improved activity than the wild-type enzyme (Fig. 6C and Table 3).

**Table 3:** Kinetic parameters of the engineered CYP76AH24 variants.

Oxidized carbon	Substrate	Enzyme	*k*_cat_ (min^−1^)	*K*_M_ (μM)	*k*_cat_/*K*_M_ (× 10^3^ min^−1^ M^−1^)	Source
C-12	abietatriene	CYP76AH24	5.51 ± 0.71	20.62 ± 6.12	267.2	ref. 19
		CYP76AH24(V296L)	6.24 ± 0.93	20.03 ± 6.97	311.5	this study
		CYP76AH24(F112L)	9.69 ± 1.31	15.67 ± 5.53	618.4	this study
C-11	ferruginol	CYP76AH24	6.11 ± 0.45	45.84 ± 9.37	133.3	ref. 19
C-2*α*	ferruginol	CYP71BE52	0.04 ± 0.002	3.96 ± 0.72	10.1	ref. 19
C-20	ferruginol	CYP76AK6	1.43 ± 0.21	20.81 ± 7.76	68.7	ref. 19

### Establishing specific pisiferic acid and salviol production in yeast cells

We analyzed the effect of the identified variants on salviol and pisiferic acid production in yeast. For pisiferic acid, cells expressing CYP76AH24(V296L) or CYP76AH24(F112L) were co-transformed with the second CYP involved in carnosic acid pathway, CYP76AK6. In both cases, the product profile of the engineered yeast cells revealed a shift towards pisiferic acid formation (Table S2 and Fig. 4A). In the case of CYP76AH24(V296L), this shift was smaller and carnosic acid remained as a main product, suggesting that the low amounts of 11-hydroxy-ferruginol produced by CYP76AH24(V296L) were sufficient to sustain significant levels of carnosic acid, presumably due to the preference of CYP76AK6 for this substrate^19^. However, when CYP76AH24(F112L) was used (Fig. 6A), carnosic acid production was completely abolished (Fig. 4A). Thus, the CYP76AH24(F112L) variant enabled the complete shift from carnosic acid to pisiferic acid production in yeast. Coupled with the metabolic interventions described for the optimization of carnosic acid production in yeast, final pisiferic acid titers reached 2.65 mg/L, which represents a total improvement of 24-fold over the basic strain (Table S2).

For salviol production, cells expressing CYP76AH24(V296L) or CYP76AH24(F112L) were co-transformed with the ferruginol 2α-hydroxylating enzyme CYP51BE52. Using both variants, a noticeable improvement in salviol titers was observed (Fig. 4B and Table S2). In the case of CYP76AH24(V296L), although low levels of ferruginol and miltiradiene oxidation at C-11 could still be observed, suppression of these side pathways increased salviol yields by almost 3.5-fold (Fig. 4B and Table S2). However, when CYP76AH24(F112L) was used, 11-hydroxy-ferruginol and 11-keto miltiradiene production was completely abolished and the salviol titer was improved significantly, reaching ~15 mg/L and a total of 14-fold improvement from the initial yield (Table S2 and Fig. 4B).

## Discussion

Many plant specialized metabolites can be obtained in sufficient amounts by extraction from their natural sources (e.g. carnosic acid, artemisinin, tanshinones, forskolin). In this case, the molecule of interest is often also the main product of the corresponding biosynthetic pathway. However, many more compounds with potential industrial application are only produced in low amounts or in complex mixtures. Obtaining access to these molecules requires navigating through the chemical complexity created by the inherent biosynthetic plasticity of secondary metabolism. The two molecules studied in this work, pisiferic acid and salviol, have been reported to have interesting biological properties but their limited availability prevented further characterization. Pisiferic acid, was found to have antibacterial and antifungal properties^30, 31^, while salviol, found in the bioactive extract of *S. miltiorrhiza* roots^25, 26^, has not yet been evaluated for its biological properties. However, several other compounds also found in the same extract, such as tanshindiol B and C or tanshinol, that had for long been overlooked due to their low abundance, have recently been found to have potent bioactivity^38–40^. Establishing pisiferic acid and salviol production in yeast will allow the thorough evaluation of their biological activity and the synthesis of derivatives.

Efforts in engineering microorganisms for the production of high-value terpenoids have achieved significant yield improvements by enhancing precursor flux, rerouting specific endogenous pathways, or balancing enzyme levels in the engineered pathway^19, 41, 42^. However, biosynthetic enzyme properties, such as low catalytic activity towards desired substrates or functional promiscuity resulting in branched pathways and reduced metabolic flux, frequently hamper metabolic engineering efforts. Pioneering work by Leonard and co-workers showed that combining protein and metabolic engineering can address these challenges^43^. By engineering functional mutations in two key enzymes, the GGPP synthase and levopimaradiene synthase (LPS), a 2600-fold improvement in levopimaradiene yield was achieved. A main challenge in production of complex terpenoids is the requirement of modifying enzymes, such as CYPs. Previous protein engineering efforts of CYP enzymes include the improvement of regio-, stereo- and chemoselectivity of CYP102A1 towards different terpene substrates including limonene and valencene^44^, the identification of a critical residue for the second oxidation step of amorpha-4,11-diene by CYP71AV1^45^, and the change of regio-selectivity of CYP720B1 towards miltiradiene^11^.

This study is the first example of engineering the specific production of minor pathway products. Using a protein engineering approach, we limited the promiscuity of CYP76AH24, the enzyme residing at the branching point of carnosic acid, pisiferic acid and salviol biosynthesis (Fig. 1). Two mutations, V296L and F112L, were identified to reduce or abolish, respectively, the ability of the enzyme to catalyze oxidation at C-11, achieving the biosynthetic shift from 11-hydroxy or catechol-type compounds to 12-hydroxy-type compounds. Introducing these variants into yeast enabled production of the minor compound pisiferic acid and improved production of salviol by avoiding the draining of precursors by the formation of undesirable side-products. Thus, protein engineering can be combined with metabolic engineering to facilitate steering through the complex landscape of plant specialized metabolism.

Even in the improved yeast strains, significant accumulation of ferruginol is observed (Table S2). Kinetic analysis shows that this is likely due to the lower catalytic efficiency of CYP76AK6 and CYP71BE52 compared to CYP76AH24 (Table 3; compare the reaction of CYP76AH24 with abietatriene with the oxidation of C-20 or C-2 of ferruginol by CYP76AK6 or CYP71BE52, respectively). These parameters have been determined using yeast microsomal preparations of the three CYPs and could reflect the different efficiency of these CYPs in the yeast system. In the plant, accumulation of ferruginol is not observed^21^, suggesting that in this environment either the specific activity of these CYPs are different, or the relative levels of the three enzymes are adjusted for optimal pathway function. In this sense, further improvement of the current yeast platform could involve balancing of the CYP levels using variable promoter strength or gene copy number.

Our results reveal that a single-residue substitution (V296L or F112L) in the key enzyme CYP76AH24 is sufficient to shift the output of the whole pathway from carnosic acid to pisiferic acid or salviol. In the context of plant adaptation, these findings suggest a molecular basis for the rapid evolution of terpene biosynthetic pathways in response to environmental cues. Mechanistically, the change in specificity observed in the present study requires only a minor flip or rotation of the substrate in order to position C-11 further away from the heme center while maintaining C-12 in proximity. Indeed, the two identified mutations (V296L or F112L) are predicted to have a relatively small impact on the active site contour. Interestingly, they can be achieved with a single-nucleotide substitution. Similar one-residue specificity switches have been reported for several terpene synthases^46–48^ and CYPs^11, 22, 44, 45^. Taken together, these observations suggest that a change in a single amino acid, close or even possibly further away from the substrate binding site, can result in a sufficient alteration in geometry or dynamics to achieve a significant change in regio-specificity. It can be envisioned that following a gene duplication event, a limited number of nucleotide substitutions would be sufficient to rapidly evolve a new biosynthetic activity in response to a change in the environment. If this enzyme sits in a central node, such as CYP76AH24 in this case, the outcome could redirect the whole pathway towards a new set of structurally related compounds. Thus, this synthetic biology approach highlights the genetic and molecular mechanisms of the phenotypic plasticity of plant natural products biosynthesis and opens up the way of controlling inherent traits for the selective production of high-value compounds.

## Materials and Methods

### Yeast media

Complete Minimal (CM) medium, composed of 0.13% (w/v) dropout powder (all essential amino acids), 0.67% (w/v) Yeast Nitrogen Base w/o AA (Y2025, US Biologicals) and 2% D-( + )-glucose monohydrate (16301, Sigma) was used for cultivation of yeast cells. For galactose-based medium, glucose was substituted with 2% D-( + )-galactose (G0625, Sigma) and 1% raffinose pentahydrate (R1030, US Biological).

### Chemicals and enzymes

Standard compounds carnosic acid (Fluka, 91209) and sclareol (VIORYL SA. Athens, Greece) were used. Other standards, such abietatriene, miltiradiene, pisiferic acid and salviol were obtained from our in-house collection, isolated from natural sources and characterized by NMR analyses. PCR amplifications were performed using Phusion High-Fidelity DNA polymerase (New England BioLabs, M0530) and MyTaq DNA polymerase (BIO-21105, Bioline). Restriction enzymes from New England BioLabs were used for cloning purposes. NucleoSpin Plasmid Kit (740588, Macherey-Nagel) was used for plasmid DNA purification. QIAquick Gel Extraction Kit (#28704, Qiagen) was used for gel extraction and DNA purification.

### Gene cloning and expression vectors

#### Cloning of Salvia pomifera cytochrome b5

The open reading frame of Cyt*b*_5_ was amplified from *S. pomifera* glandular trichome cDNA using primers SpCypB5-BamHI and SpCypB5-XhoI (Table S3) which incorporated *Bam*HI and *Xho*I flanking restriction sites at the 5′ and 3′ of the ORF, respectively. The PCR product was purified and cloned into pCRII-TOPO TA, according to the manufacturer’s instructions (Invitrogen). For expression from low copy number vector under the constitutive promoter P_TPI1_, the ORF of SpCyt*b*_5_ was digested with *Bam*HI and *Xho*I from pCRII-TOPO vector and ligated to pYX143 (P_TPI1_, *LEU2*, cen) digested with the same enzymes. For expression from high copy number plasmid under the strong inducible promoter P_GAL10_, the SpCyt*b*_5_ ORF was amplified from the pCRII-Topo vector using primers Cyt*b*_5_-NotI-5 and Cyt*b*_5_-SacI-3 (Table S3) to introduce 5′-*Not*I and 3′-*Sac*I flanking restriction sites. The PCR product was purified and cloned into the pCRII-TOPO TA and subsequently digested with *Not*I and *Sac*I and ligated to pESC-Leu and pESC-LEU/CPR2 vectors (Agilent Technologies) linearized with *Not*I and *Sac*I.

#### Construct pYX143/CPR2

The ORF of *Populus trichocarpa* NADPH-cytochrome P450 reductase (XM_006381734) was PCR amplified from plasmid pESC-LEU/CPR2-PtAO kindly provided by Prof. J. Bohlmann (University of British Columbia, Canada) and primers CPR2-MfeI and CPR2-XhoI (Table S3) which incorporated *Mfe*I and *Xho*I flanking restriction sites at the 5′ and 3′ of the ORF respectively. The PCR product was purified and cloned into the pCRII-TOPO TA. The insert was digested with *Mfe*I and *Xho*I and ligated to pYX143 (P_TPI1_, *LEU2*, cen) digested with *Eco*RI and *Xho*I. For expression from a high copy number plasmid under the strong constitutive promoter P_TDH3_, the CPR2 insert digested with *Mfe*I and *Xho*I was ligated into pWTDH3 digested with *Eco*RI and *Xho*I.

#### Cloning M1-M2a fusions with different GS linkers

The constructs pYES/SfCDS-ERG20(F96C) containing different GS linkers were generated as previously described^19^ using primers SfCDS-BamHI-5 and SfCDS-5xGS-MfeI or CDS-GSG-MfeI (Table S3).

#### Cloning M3a and M3b into pESC-Trp vector

CYP76AK6 was PCR amplified from pESC-Leu::CPR2-CYP76AK6 construct^19^ using primers Sp76-5-BamHI and Sp76-5-SalI (Table S3) which incorporated *Bam*HI and *Sal*I restriction sites. Following PCR product purification and TOPO cloning, the insert digested with *Bam*HI and *Sal*I was ligated into pESC-Trp vector (Agilent Technologies) linearized with the same enzymes. This cloning strategy introduced CYP76AK6 under the control of P_GAL1_ promoter. CYP76AH24 was digested from pESC-Leu::CPR2-CYP76AH24 construct^19^ using *Eco*RI and *Not*I restriction enzymes and ligated into the previously generated construct pESC-Trp::CYP76AK6 liniarized with *Eco*RI and *Not*I to allow gene expression from P_GAL10_ promoter.

### Generation of AM141 strain

To develop the yeast integration cassette overexpressing the *S. pomifera* Cyt*b*_5_, the ORF was excised from pCRII/SpCypB5 using *Bam*HI and *Xho*I, and ligated to COD7 plasmid^49^ linearized in the same manner. The integration cassette COD7/Sp Cyt*b*_5_ was PCR amplified using primers YAL065C-5COD7 and YAL065C-3COD7 (Table S3) which target the cassette to the 3′UTR of YAL065C gene. The purified PCR fragment was transformed into the AM119-4 yeast strain and successful integration was selected in glucose-CM plates lacking histidine. Proper integration was validated by PCR on genomic DNA isolated from several growing colonies using primers YAL065C UP (Table S3) which is located at the upstream genomic region and TDH3-R conf which binds on the integration cassette.

### Mutagenesis

Site-directed mutagenesis of CYP76AH24 was performed as described in ref. 50 using the Quickchange method (Agilent). Introduction of nucleotide changes were performed by the degenerate primers described in Table S3.

### Yeast strain cultivation, terpene quantification, and extraction from yeast cells

Yeast cells were cultivated as previously described^11^. Cultures grown until OD_600_ = 0.7–1 were switched to galactose-raffinose based selective growth medium (10 mL) for expression of the SpCDS-Erg20p(F96C) fusion, CPR2, Cyt*b*_5_ and CYP76AH24, CYP76AK6 or CYP71BE52 under the galactose-inducible promoters P_GAL1_ or P_GAL10_. For production, cultures were cultivated for 2 days. Terpene extraction was performed by 10% dodecane overlay or solvent (pentane or ethyl acetate) extraction using aliquots of 1 mL cultures. For GC-MS analysis of the oxygenated diterpenoids, carnosic acid, pisiferic acid and salviol, solvent extracts were derivatized prior to GC-MS analysis using the following procedure described in ref. 19. The compounds produced were quantified by GC-FID analysis of the solvent extracts, as described in ref. 46.

### Microsomal protein preparation and cytochrome P450 quantification

Yeast cultures of 250 mL were used to prepare microsomal proteins as previously described^51^. An additional final ultracentrifugation step at 100,000 *g* for 60 min was performed. The quantification of CYPs was carried out as described by Omura and Sato^52^, by measuring the spectroscopic difference at 450 nm resulting from the binding of CO to the reduced form of CYP enzymes. The extinction coefficient 91 mM^−1^ cm^−1^ was used. For background correction, microsomal preparations from cells carrying CPR2 but an empty vector with respect to CYP genes were used (negative control).

### *In vitro* enzymatic assay and kinetic analysis

The kinetic parameters of the CYP76AH24 variants were determined as previously described^11^ using varying concentrations (1–75 μM) of each substrate. The enzymatic reactions were incubated with mild shaking at 30 °C for 3 h and terminated by extraction with 100 μL of decane or ethyl acetate containing 10 μg/mL sclareol as internal standard. Extracts (2 μL) were analyzed by GC-MS. All assays were carried out in duplicates.

GC-MS analysis was carried on a DB-5 column using helium as a carrier gas with a constant velocity of 40 cm/sec. Different temperature programs were used according to the extraction procedure. All samples (2 µl) were injected in splitless mode. Samples from dodecane culture overlays were analyzed using the temperature program described in reference^32^. Briefly, after a 3 min hold at 60 °C, the temperature was increased to 240 °C with a gradient of 15 °C/min, concluding with a 10 min hold at 200 °C. The scan range was from 35 to 450 *m/z*. For the analysis of ethyl acetate or pentane extracted and TMS-derivatized samples, the temperature program initiated at 60 °C, and the temperature was increased to 200 °C with a rate of 15 °C/min, where it was held for 10 min, and then raised at 15 °C/min to 290 °C and held for 20 min. MS data from 50 to 550 *m/z* were collected during the temperature ramp^19^.

## Electronic supplementary material

Supplementary Info
