# Supplementary material for: Overcoming the plasticity of plant specialized metabolism for selective diterpene production in yeast
Source: Sci Rep. 2017 Aug 18;7:8855. doi: 10.1038/s41598-017-09592-5 (PMC5562805; doi:10.1038/s41598-017-09592-5)
Supplement: Supplementary file 1 — Supplementary Info [file 41598_2017_9592_MOESM1_ESM.doc]

**Supplementary Information**

Overcoming the plasticity of plant specialized metabolism for selective diterpene production in yeast

[Codruta Ignea](http://www.ncbi.nlm.nih.gov/pubmed/?term=Ignea C%5Bauth%5D)^1,2^, Anastasia Athanasakoglou^1,2^, Aggeliki Andreadelli^3^, Maria Apostolaki^4^, Minas Iakovidis^4^, Euripides G. Stephanou^4^, [Antonios M. Makris](http://www.ncbi.nlm.nih.gov/pubmed/?term=Makris AM%5Bauth%5D)^3^ and [Sotirios C. Kampranis](http://www.ncbi.nlm.nih.gov/pubmed/?term=Kampranis SC%5Bauth%5D)^1,2*^

^1^Department of Plant and Environmental Sciences, University of Copenhagen, Thorvaldsensvej 40, 1871 Frederiksberg C, Denmark

^2^Department of Medicine, University of Crete, P.O. Box 2208, Heraklion 71003, Greece

^3^Institute of Applied Biosciences – Centre for Research and Technology Hellas (INAB-CERTH), P.O. Box 60361, Thermi 57001, Thessaloniki, Greece

^4^Department of Chemistry, University of Crete, P.O. Box 2208, Heraklion 71003, Greece

^*^Corresponding author: soka@plen.ku.dk

**DNA sequence of SpCyt*b_5_* ORF**

atggcgaaatctcacacatttgaggaggtcgcgaagcacaacaagactaaggattgctggctcattatcagcggaaaggtttatgatgtgaccccattcatggaggatcatcctggaggtgatgaagttttgctgtccgcgaccggaaaagacgctaccaatgattttgaagatgttgggcacagcgattctgctcgggagatgatggacaaatacttcatcggtgagatagacatggcaacggttcccctaaaacgaagctacatagctccacaacaaccatcatacaatccagacaagaccccagagtttgtgatcaagatcttgcagttcctcgtgcctctcctgatcttgggattggcatttgctgtccggctctacaccaaagagaaatga

**Table S1. Carnosic acid production in yeast under different levels of CPR and cyt*b5*** expression.

| **CPR expression** | | **Cyt*b_5_* expression** | | **Carnosic acid (mg/L)** |
| --- | --- | --- | --- | --- |
| **Promoter** | **Copy number** | **Promoter** | **Copy number** |
| P_TPI1_ | ARS/CEN | P_TDH3_ | *::3’YAL065* | 18.09 ± 0.82 |
| P_TPI1_ | ARS/CEN | P_TPI1_ | ARS/CEN | 12.32 ± 1.02 |
| P_TPI1_ | ARS/CEN | P_TDH3_ | 2μ | 11.21 ± 0.73 |
| P_TDH3_ | 2μ | P_TDH3_ | *::3’YAL065* | 10.80 ± 1.20 |
| P_GAL1_ | 2μ | P_TDH3_ | *::3’YAL065* | 9.15 ± 0.50 |
| P_GAL1_ | 2μ | P_TPI1_ | ARS/CEN | 7.63 ± 0.21 |
| P_TDH3_ | 2μ | P_GAL10_ | 2μ | 7.05 ± 0.72 |
| P_TDH3_ | 2μ | P_TPI1_ | ARS/CEN | 6.74 ± 0.36 |
| P_GAL1_ | 2μ | P_GAL10_ | 2μ | 5.69 ± 0.14 |

**Table S2. Compound profile in engineered yeast cells**

| Enzymatic pathway | 11-keto miltiradiene (mg/L) | ferruginol  (mg/L) | 11-hydroxy-ferruginol  (mg/L) | pisiferic  acid  (mg/L) | carnosic  acid  (mg/L) | salviol  (mg/L) | 11β-hydroxy-manoyl oxide (mg/L) |
| --- | --- | --- | --- | --- | --- | --- | --- |
| CYP76AH24wt | 13.5 ± 0.98 | 25.6 ± 1.02 | 19.7 ± 2.12 | 0.11 ± 0.05 | 1.0 ± 0.12 | 1.1 ± 0.02 | 2.3 ± 0.14 |
| CYP76AH24(V296L) | 0.0 ± 0.0 | 32.8 ± 1.35 | 2.5 ± 0.08 | 0.85 ± 0.03 | 0.2 ± 0.01 | 8.7 ± 1.02 | 0.1 ± 0.01 |
| CYP76AH24(F112L) | 0.0 ± 0.0 | 48.8 ± 0.75 | 0.0 ± 0.0 | 2.65 ± 0.05 | 0.0 ± 0.0 | 15.5 ± 1.53 | 0.0 ± 0.0 |

**Table S3. List of primers used in this study**

| **Gene Name** | **Primer** | **Sequence** |
| --- | --- | --- |
| CYP76AH24 | FS112HL | gacaagatctcgatggggCWTctccccgtcggggc |
| CYP76AH24 | FS296FLI | ctcatgctggacttgttcHttggaggatctgaaactagcac |
| CYP76AH24 | FS477HL | cccttcgcaccgcaWgcccaaacaacacacctttg |
| CYP76AH24 | MyGAL10 | gatatgtatatggatatgtatatggtgg |
| CYP76AH24 | MyADH1 | gagaaagcaacctgacctacagg |
| CYP76AK6 | Sp76-5-BamHI | ggatccatgcaagttctcatccttctttctctggccttcctagca |
| CYP76AK6 | Sp76-5-SalI | gtcgactcaaactttgatgggaatagctcttagggggattttcttct |
| SfCDS | SfCDS-BamHI-5 | ggatccatggcgcccctgacttgc |
| SfCDS | CDS-GSG-MfeI | caattggccgctaccgatacgaccggtccaaagagtactt |
| SfCDS | CDS-5XGS-MfeI | caattggctaccgctgccgctaccgctgccgctacctacgaccggtccaaagagtactt |
| Cyt*b_5_* | CytB5-NotI-5 | gcggccgcatggcgaaatctcacacatttg |
| Cyt*b_5_* | CytB5-SacI-3 | gagctctatttctctttggtgtagagccg |
| Cyt*b_5_* | SpCypB5-BamHI | ggatccatggcatcagatccgaaatctcacacatttgag |
| Cyt*b_5_* | SpCypB5-XhoI | ctcgagtcatttctctttggtgtagagccggacagca |
| CPR2 | CPR2-MfeI | caattgatgcaatcatcaagcagctcgatgaaagtgtcac |
| CPR2 | CPR2-XhoI | ctcgagttaccatacatcacgcagatacctgccattc |
| YAL065c | YAL065c-5COD7 | acgcacggcatcatcttatattaataattctatcatcacgcttatagtgtttaccagttcgagtttatcattatc |
| YAL065c | YAL065c-3COD7 | taatgcaaagccgaatagttaggctaaaaatgtactcttagacatttaaaaaggtggatctgatatcaccta |
| YAL065c | YAL065c UP | ctgttgaccaataatggcataagtgt |
| TDH3 | TDH3-R | tagtggatgccaggaataaactgttcac |
